# Small protein B upregulates sensor kinase *bvgS* expression in *Aeromonas veronii*

**DOI:** 10.3389/fmicb.2015.00579

**Published:** 2015-06-16

**Authors:** Zhu Liu, Peng Liu, Shuanshuan Liu, Haichao Song, Hongqian Tang, Xinwen Hu

**Affiliations:** ^1^Hainan Key Laboratory for Sustainable Utilization of Tropical Bioresources, College of Agriculture, Hainan UniversityHaikou, China; ^2^Department of Biology, College of Sciences, Shantou UniversityShantou, China

**Keywords:** SmpB, BvgS, upregulation, *trans*-translation, *A. veronii*

## Abstract

Earlier studies reveal that Small protein B (SmpB), a class of well-conserved tmRNA-binding proteins, is essential for the *trans*-translation process, which functions as a system for translation surveillance and ribosome rescue. Here, we report a previously unrecognized mechanism by which SmpB alone positively regulates the expression of a sensor kinase, BvgS, in *Aeromonas veronii*. A reporter plasmid was constructed in which the promoter of *bvgS* was used to control the expression of the enhanced green fluorescent protein (eGFP) gene. When the reporter plasmid was co-transformed with a SmpB expression construct into *E. coli*, the relative fluorescence intensity increased about threefold. Transformation with a truncated form of *smpB* gene showed that the C-terminus had little effect, while N-terminus unexpectedly increased eGFP production. Next, a series of SmpB mutants were generated by site-directed mutagenesis. When the mutants SmpB (G11S) or SmpB (E32AG) was used in the experiment, eGFP expression dropped significantly compared with that of wild type SmpB. Further, purified SmpB was shown to bind the promoter regions of *bvgS* in the agarose gel retardation assay. Quantitative RT-PCR analysis showed that eGFP transcript levels increased approximately 25-fold in the presence of SmpB. Likewise, *smpB* knockout decreased *bvgS* transcripts significantly in *A. veronii*, and also displayed a reduced capability in salt tolerance. Collectively, the data presented here will facilitate a deeper understanding of SmpB-mediated regulatory circuits as a transcriptional factor in *A. veronii*.

## Introduction

*Trans*-translation mediated by tmRNA and SmpB is a vital quality control system which rescues stalled ribosomes and degrades incomplete nascent proteins for recycling in bacteria. This ensures the availability of ribosomes for protein synthesis and prevents the accumulation of dysfunctional proteins (Keiler, 2008). It has been reported that *trans*-translation is widespread in eubacteria, and required for stress tolerance, growth, and virulence (Moore and Sauer, 2007). Mutations in the *trans*-translation system render pathogen *Francisella tularensis* vulnerable to stress and become avirulent in mice (Svetlanov et al., 2012). The targeted inactivation of tmRNA and SmpB increases sensitivities to antibiotics, and oxidative and nitrosative stresses in *Yersinia pseudotuberculosis* (Okan et al., 2006). Deletion of *tmRNA* or *smpB* genes causes a defect in the timing of DNA replication in *Caulobacter crescentus* (Keiler and Shapiro, 2003). However, it is hard to explain why *trans*-translation mutants in various species have diverse phenotypes of physiology since tmRNA and SmpB appear to be conserved throughout the bacterial kingdom. Similarly, the system for translational surveillance and ribosome rescue is universal and performs uniform functions to all genes in eubacteria, it does not clarify why it affects prominently on virulence, growth, and viability.

According to the *trans*-translation mechanism, tmRNA and SmpB exert identical influences on the tolerances to a variety of antibiotics and stresses in *E. coli*. As a matter of fact, SmpB deficiency increases susceptibility to antibiotics compared with tmRNA deficiency (Li et al., 2013). Besides, tmRNA can act as an antisense RNA to regulate pigment synthesis (Liu et al., 2010). All these results suggest that SmpB and tmRNA might play additional roles outside the *trans*-translation system.

The Gram-negative bacteria *Aeromonas veronii* is the causative agent which initiates mass mortalities in fish species, and consequently leads to catastrophically economic losses in the fish-farming industry (Li et al., 2011; Reyes-Becerril et al., 2014). Earlier genetic evidences show that many prokaryotic genes evolve in the vicinity due to their functional synergism. Therefore, the gene *bvgS*, which positions close to the gene *smpB* is investigated as a potential SmpB protein target in *A. veronii*. The gene *bvgS* encodes a histidine kinase and tandem expresses with its cognate response regulator (RR), and performs as a sensor in the two-component signal transduction systems (TCSs). In bacteria, activation of TCSs eventually results in the alterations of a number of cellular processes including bacterial growth, virulence, motility, and biofilm formation (Bekker et al., 2006; Hett and Rubin, 2008; Clarke, 2010; Wu et al., 2015). To date, neither *A. veronii* nor the closely related *Aeromonas* species are studied on BvgS/RR TCS, while there are widely researched in *Bordetella pertussis.* Upon activation, BvgS autophosphorylates a conserved histidine and then transfers the phosphoryl group to its cognate regulator, which subsequently propagates the signals to modulate downstream gene expressions (Prajapat and Saini, 2013).

In present study, we demonstrated that SmpB contributed to the enhancement of *bvgS* expression by binding to the *bvgS* promoter as a transcriptional regulator. The presented results not only deepen the understanding of SmpB function outside *trans*-translation, but also help to find new therapeutic targets for pathogenic prevention.

## Materials and Methods

### Plasmid Constructions

Plasmids used in this work were given in **Table 1**, and all used primers were listed in Supplementary Table S1. In PCR reaction, the thermocycle parameters were as follows: 98°C for 2 min, followed by 98°C for 30 s, 60–65°C for 30 s and 72°C for 1 kb/min in a total of 35 cycles. For construction of pDH114, the *bvgS* promoter region, which included 200 bp upstream and 27 bp downstream of the start codon, was amplified from genomic DNA of *A. veronii* C4 by PCR using primers F1/R1. The eGFP fragment was amplified from pEGFP-C2 with primers F2/R2 (Qing et al., 2014). Purified and mixed PCR products (10 μl each, 500 ng/μl) were used in an overlap extension PCR (Heckman and Pease, 2007) using primers F1/R2, which contain a *Bgl* II and *Eag* I site, respectively, at the 5^′^-end. The resulting 1 kb DNA fragment was excised from a 1% agarose gel, purified and digested with *Bgl* II and *Eag* I, and then inserted into plasmid pDH113 (pBR322 origin and ampicillin resistance), a generous gift from Dr. Buskirk’s lab (Watts et al., 2009).

**Table 1:** Plasmids used in this study.

Plasmid	Description	Reference
pEGFP-C2	Enhanced green fluorescent protein (eGFP) expression vector.	Qing et al. (2014)
pDH113	pBR322 origin, ampicillin resistance, SmpB expression.	Watts et al. (2009)
pDH210	p15A origin, terramycin resistance, tmRNA transcription.	Watts et al. (2009)
pET-28a (+)	T7 promotor, kanamycin resistance, expresses His Tag.	Merck KGaA, Darmstadt, Germany
pDH114	pBR322 origin, ampicillin resistance, includes BvgS promoter and expresses 9-residue at the N terminus of BvgS with full length eGFP.	This study
pBT	p15A origin, chloramphenicol resistance, includes lac-UV5 promoter and cI ORF	Keysight Technologies, CA, USA
pDH211	p15A origin, terramycin resistance, includes *smpB* promoter and the whole smpB and tmRNA encoded region.	This study
pDH212	p15A origin, terramycin resistance, includes *smpB* promoter and the whole smpB encoded region.	This study
pDH213	p15A origin, terramycin resistance, includes only *smpB* promoter.	This study
pDH214	p15A origin, terramycin resistance, includes GST promoter and the whole GST encoded region.	This study
pET-SmpB	T7 promotor, kanamycin resistance, expresses *smpB* with His Tag.	This study
pBT-SmpB	p15A origin, chloramphenicol resistance, expresses *smpB* with cI.	This study
pBT-SmpB N34	pBT-SmpB derivative, deletes 34-residue at the N terminus of SmpB.	This study
pBT-SmpB C30	pBT-SmpB derivative, deletes 30-residue at the C terminus of SmpB.	This study
pDH212(SmpB-N1)	pDH212 derivative, substitues first residue to stop codon at the N terminus of SmpB.	This study
pDH212(SmpB-N35)	pDH212 derivative, substitues 35th residue to stop codon at the N terminus of SmpB.	This study
pDH212(SmpB-C33)	pDH212 derivative, substitues 33rd residue to stop codon at the C terminus of SmpB.	This study
pBT(SmpB-G11S)	pBT-SmpB derivative, mutates G11S to AA.	This study
pBT(SmpB-T14I)	pBT-SmpB derivative, mutates T14I to AA.	This study
pBT(SmpB-F26I)	pBT-SmpB derivative, mutates F26I to AA.	This study
pBT(SmpB-E32AG)	pBT-SmpB derivative, mutates E32AG to AAA.	This study
pBT(SmpB-G133K)	pBT-SmpB derivative, mutates G133K to AA.	This study
pBT(SmpB-D138KR)	pBT-SmpB derivative, mutates D138KR to AAA.	This study
pBT(SmpB-K152)	pBT-SmpB derivative, mutates K152 to P.	This study
pRE112	Suicide plasmid for gene knock out, chloramphenicol resistance, includes a conditional R6K ori requiring the π protein for replication, expresses *sacB* for sucrose selection.	Edwards et al. (1998)
pRE-Δ*smpB*	pRE112 derivative for *smpB* knock out in *Aeromonas veronii* C4, chloramphenicol resistance.	This study
pRE-Δ*tmRNA*	pRE112 derivative for *tmRNA* knock out in *A. veronii* C4, chloramphenicol resistance.	This study

For construction of pDH212, a 1 kb DNA fragment containing about 500 bp promoter and 489 bp encoding region of *smpB* gene was amplified from genomic DNA of *A. veronii* C4 with primers F4/R4 which carried a *Nco* I and *Xho* I site, respectively, at the 5^′^ end. The fragment was cut with *Nco* I/*Xho* I and inserted into plasmid pDH210 (p15A origin and terramycin resistance), which was generously provided by Dr. Buskirk (Watts et al., 2009). The plasmids pDH211, pDH213, and pDH214 were constructed as the controls for evaluation of SmpB function using the same strategies.

For construction of pET-SmpB, a 486-bp DNA fragment containing the encoding region of SmpB was amplified from genomic DNA of *A. veronii* C4 with primers F7/R7, containing a *Nco* I and *Xho* I site, respectively, at the 5^′^-end. The fragment was cut with *Nco* I/*Xho* I and inserted into the expression vector pET-28a (+) (Merck KGaA, Darmstadt, Germany).

For construction of pBT-SmpB, the DNA fragment encoding SmpB protein was amplified from genomic DNA of *A. veronii* C4 with primers F8/R8, containing an *Eco*R I and *Bgl* II site, respectively, at the 5^′^-end. The fragment was cut with *Eco*R I/*Bgl* II and inserted into pBT (Keysight Technologies, Santa Clara, CA, USA). The truncation vectors pBT-SmpB ΔN34 was created using pBT-SmpB as template and F9/R9 as primer pair. Likewise, pBT-SmpBΔC30 was constructed with the replacement of the primer pair as F10/R10.

For construction of pRE-ΔSmpB, both 400 bp upstream and downstream DNA fragments of SmpB target were amplified from genomic DNA of *A. veronii* C4 by PCR using primers F11/R11 and F12/R12, respectively. Purified and mixed PCR products (10 μl each, 500 ng/μl) were used in an overlap extension PCR (Heckman and Pease, 2007) using primers F11/R12, which contain a *Kpn* I and *Sac* I site, respectively, at the 5^′^-end. The resulting 800 bp fusion DNA fragment was excised from a 1% agarose gel, purified and digested with *Kpn* I and *Sac* I, and then inserted into plasmid pRE112, which was generously provided by Dr. Kangsheng Li at Shantou University (Edwards et al., 1998).

For construction of pRE-Δ*tmRNA*, 400 bp upstream DNA fragments of tmRNA target were amplified from genomic DNA of *A. veronii* C4 by PCR using primers F14/R14 containing a *Hin*d III*/Xba* I site, respectively, at the 5^′^-end. Consistently, 400 bp upstream DNA fragments of tmRNA were amplified using primers F15/R15 containing *Xba* I/*Xma* I sites at the 5^′^-end. Each fragment was cut with appropriate restriction endonucleases and inserted into pRE112 (Edwards et al., 1998).

Site-directed mutagenesis was performed according to the protocol provided by QuickChange Kit (Qiagen, Shenzhen, China). For constructing pDH212(SmpB-N1), in which first residue was substituted to stop codon at the N-terminus, pDH212 was diluted with ddH_2_O to a concentration of 400 pg/μl, and used as the template. In the PCR reaction, 1 μl of DNA template, 1 μl primer F17 or R17 (10 mM), 25 μl PrimeSTAR mix (Takara, Dalian, China) were included in the 50 μl volume. After running for 20 cycles, the reactions were paused, and subsequently both PCR products were combined for an additional 10 cycles. At the end, PCR product was purified and digested with *Dpn* I before being transformed into *E. coli/ΔtmRNA-smpB*, in which both *tmRNA* and *smpB* were deleted (Hallier et al., 2004). The mutated plasmids were purified and confirmed by DNA sequencing (Sangon Biotech, Shanghai, China). Likewise, the pDH212 derivative mutants, pDH212(SmpB-N35) and pDH212(SmpB-C33), were obtained accordingly with primer sets F17/R18 and F19/R19. As for pBT-SmpB derivatives including pBT(SmpB-G11S), pBT(SmpB-T14I), pBT(SmpB-F26I), pBT(SmpB-E32AG), pBT(SmpB-G133K), pBT(SmpB-D138KR), and pBT(SmpB-K152), site-directed mutagenesis was executed correspondingly using pBT-SmpB as the template and BacterioMatch II two-hybrid reporter strain as the host strain.

### Construction of tmRNA and SmpB Mutants in *A. veronii* C4

Gene knockout was performed according to the procedure with modifications (Edwards et al., 1998). Briefly, pRE-Δ*smpB* was transformed into *E. coli* WM3064 (Dehio and Meyer, 1997), and the positive clone was selected by plating on LB agar containing chloramphenicol and diaminopimelic acid (Dap). pRE-Δ*smpB* was then mobilized into *A. veronii* C4 by conjugation with selected *E. coli* WM3064 clones. The transconjugants, in which plasmid pRE-*smpB* had integrated into *A. veronii* C4 chromosome through a single crossover, were screened on LB agar containing chloramphenicol and ampicillin. Allelic exchange between the chromosomal gene and the plasmid was achieved after the second crossover event. *A. veronii* Δ*smpB* mutant was selected on LB agar supplemented with 6% sucrose and ampicillin, and subsequently confirmed by PCR with primers F13/R13. For the construction of *A. veronii* Δ*tmRNA* mutant, similar procedure was performed except for the substitution of pRE-ΔtmRNA with pRE-Δ*smpB*.

### Bacterial Cell Growth

Bacterial strains used in this work were presented in **Table 2**

**Table 2:** Bacterial strains used in this study.

Strain	Description	Reference
*Escherichia coli* Δ*tmRNA-smpB*	*tmRNA and smpB* genes were deleted from*E. coli*.	Hallier et al. (2004), Watts et al. (2009)
*E. coli* BL21 (DE3)	Protein expression host	New England Biolabs, Ipswich, MA, USA
BacterioMatch II two-hybrid reporter strain	MRF^′^, Kanamycin resistance	Keysight Technologies Inc, Santa Clara, CA, USA
*E. coli* WM3064	Encodes a relaxase, a mating pair formation (MPF) complex and a type IV coupling protein in chromosome, diaminopimelic acid (Dap) auxotroph.	Dehio and Meyer (1997)
*A. veronii* C4	Wild type, ampicillin resistance, virulent to fish.	This study
*A. veronii* Δ*tmRNA*	Ampicillin resistance, *tmRNA* gene was deleted from WT.	This study
*A. veronii* Δ*smpB*	Ampicillin resistance, *smpB* gene was deleted from WT.	This study

For the preparation of fluorescence quantification, *E. coli* Δt*mRNA-smpB* transformants were grown with shaking (250 rpm) in 1 ml LB media supplemented with 50 μg/ml ampicillin and terramycin at 37°C overnight (Green and Sambrook, 2012), and then the resulting cultures were inoculated into 50 ml LB media at an initial OD_600_ of 0.05. The total amount of 4 ml of cell cultures was harvested by centrifugation at 12,000 rpm for 2 min at different time courses. The pellet was resuspended in 10 ml of 1X PBS buffer (137 mM NaCl, 2.7 mM KCl, 10 mM Na_2_HPO_4_, 2 mM KH_2_PO_4_, pH = 7.4), and the fluorescence intensity was measured.

For the preparation of qRT-PCR analysis, *E. coli* Δ*tmRNA-smpB* transformants or *A. veronii* C4 mutants were cultured overnight as described above, and then inoculated into 50 ml LB media at an initial OD_600_ of 0.05. A quantity of 3 ml of cell cultures was collected at the exponential phase or stationary phase, and used for qRT-PCR measurement.

For the preparation of SmpB production, pET-SmpB was transformed into *E. coli* BL21 (DE3). Positive clones were selected and grown at 37°C until OD_600_ reached 0.6, and then isopropyl β-D-1-thiogalactopyranoside (IPTG) was added to a final concentration of 40 μM for an additional 7.5 h. A quantity of 250 ml of cell cultures was collected for SmpB production.

For the preparation of salt resistance experiment, *A. veronii* C4 and its mutants were inoculated in LB media overnight, and then inoculated into 50 ml M9 media supplemented with 0.3 M potassium chloride (KCl) at an initial OD_600_ of 0.05. The cells were harvested after cultivation with shaking at 37°C for 24 h. Cell turbidities were measured by taking 1 ml of culture broth to perform OD_600_ readings.

### Fluorescence Detection

The collected cells were suspended in 200 μl PBS buffer, and loaded to a 96-well microtiter plate (Greiner BIOONE, Nuernberg, Germany). The intensity of eGFP fluorescence was measured at 485 nm excitation and 525 nm emission wavelengths with a fluorescence microplate reader (Infinite^®^ 200 PRO, Tecan, Shanghai, China). Each sample was provided at least in triplicate. The relative fluorescence intensity was calculated as the total fluorescence intensity divided by OD_600_ value.

### Agarose Gel Retardation Assay (AGRA)

Agarose gel retardation assay (AGRA) was performed according to the procedure with modification (Das et al., 2013). Cells transformed with pET-SmpB were cultivated as described above before being intensively sonicated. After centrifugation, the supernatant was purified by Ni-NTA affinity chromatography. The fractions containing SmpB protein were pooled and dialyzed against PBS buffer at 4°C overnight. Meanwhile, a 90-bp DNA fragment containing part of the promoter of *bvgS* was amplified using primers F27/R27. Concurrently, a 300-bp DNA fragment including the promoter region of *yafJ* neighboring *smpB* was amplified using primers F28/R28 as negative control. SmpB protein in series dilution was then incubated with about 200 ng of purified *bvgS* or *yafJ* promoter in EMSA/Shift buffer for 20 min at 25°C. Complexes were resolved in a 1% agarose gel and revealed by staining with ethidium bromide (EtBr).

### RNA Extraction, Reverse Transcription, and qRT-PCR

Cells were harvested and RNA was extracted with RNAiso Plus (TaKaRa, Dalian, China) according to the manufacturer’s instructions. The integrity of RNA was checked by agarose gel electrophoresis and quantified by spectrophotometer (NanoDrop, Wilmington, DE, USA) before it was adjusted to 800 ng/μl in all samples. Reverse transcription was implemented with the ReverTraAce qPCR RT Kit (Toyonbo, Shanghai, China). PCR was employed on a ABI Prism^®^ 7300 (ABI, New York, NY, USA) equipped with an optical fluorescent detection system using SYBR^®^ Green Realtime PCR Master Mix (Toyonbo, Shanghai, China). After normalization to the 16S rRNA, RT-PCR data were analyzed using the 2^-^^Ct^ method (Schmittgen and Livak, 2008).

### Statistical Analysis

The results were displayed as the mean plus the standard error. Statistical analysis was implemented using one-way analysis of variance (ANOVA) with SPSS version 19.0 software (IBM, Armonk, NY, USA). P values less than 0.05 represented significant differences. *P*-values less than 0.01 represented extremely significant differences (Ribas-Latre et al., 2015).

## Results

### SmpB Enhanced eGFP Production under the Control of *bvgS* Promoter

**FIGURE 1:**
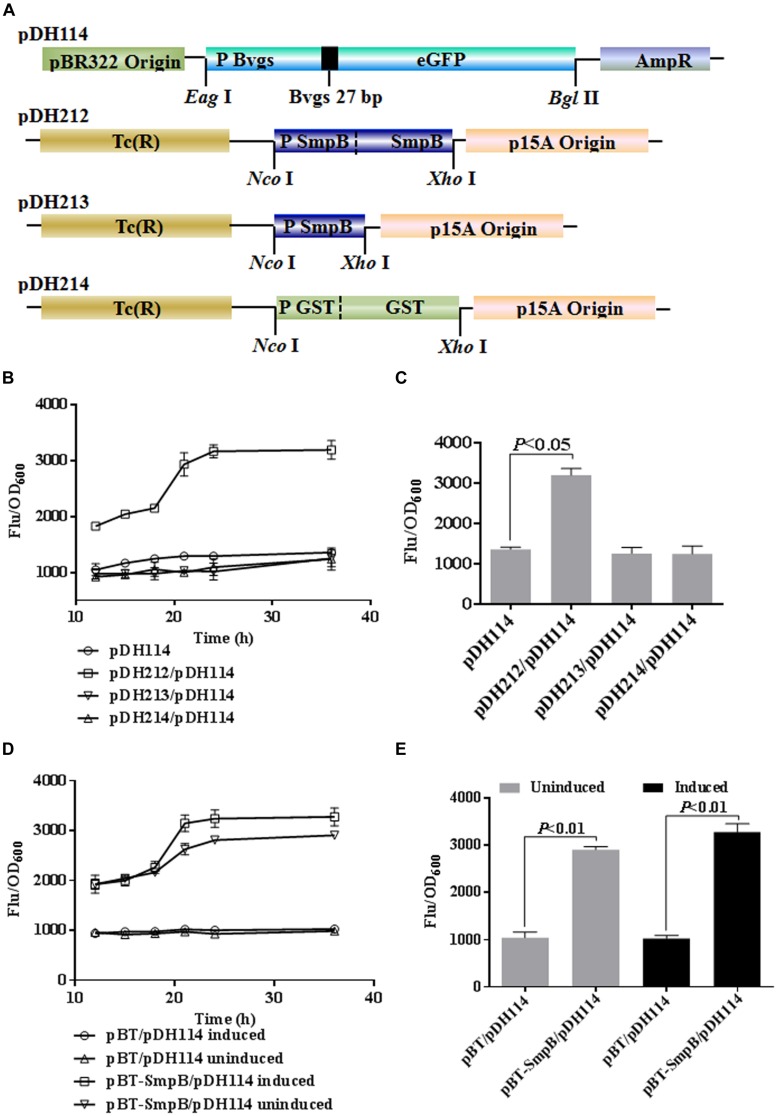
**Small protein B (SmpB) enhanced enhanced green fluorescent protein (eGFP) production under the control of *bvgS* promoter. (A)** Schematic representation of the constructs. The promoter and initial nine-residue of N-terminal BvgS were fused to eGFP, and introduced between pBR322 origin and ampicillin resistance gene to generate pDH114 by restriction sites *Eag* I and *Bgl* II. The native promoter and *smpB* gene, the native *smpB* promoter, the native promoter and encoded gene of GST were inserted between tetracyclin resistant gene and p15A origin to produce pDH212, pDH213, pDH214 using *Nco* I and *Xho* I, respectively. **(B)** Time courses of relative fluorescence in *Escherichia coli* Δ*tmRNA-smpB* co-transformed pDH114 with pDH212, pDH213, and pDH214, separately. The fluorescence of cells was measured and normalized to corresponding OD_600_. Error bars represented the SD of three independent cultures. **(C)** Column graph comparing the relative fluorescence at 24 h. **(D)** Time courses of relative fluorescence in *E. coli* two-hybrid reporter strains co-transformed pDH114 with pBT and pBT-SmpB, separately. The strains were induced by 1 mM IPTG at 37°C compared with the uninduced ones. **(E)** Column graph comparing the relative fluorescence in pBT and pBT-SmpB after 24 h IPTG treatments.

To test if SmpB could regulate the expression of *bvgS*, we constructed a series of expression cassettes (**Figure 1**). In pDH114, eGFP expression was under the control of *bvgS* promoter, while in pDH212, the expression of *smpB* gene was controlled by *smpB* promoter. As some controls, pDH213 contained *smpB* promoter only, and pDH214 carried a glutathione *S*-transferase (GST) gene under the control of its native promoter (**Figure 1A**). When pDH212, pDH213, or pDH214 was co-transformed with pDH114 into *E. coli* Δ*tmRNA-smpB*, the relative eGFP fluorescence intensity were measured at 24 h (**Figure 1B**), showing that cells carrying *smpB* expression cassette were significantly brighter than the controls (*p* < 0.05), illustrating SmpB could upregulate eGFP production under the control of *bvgS* promoter (**Figure 1C**). Analogously, when pDH114 and pBT-SmpB which carrying *smpB* expression under the control of lac-UV5 promoter were co-introduced into *E. coli* two-hybrid reporter strain, SmpB protein was found again to increase the fluorescent densities (**Figures 1D,E**).

### N- and C-Terminal Regions of SmpB were Necessary for Enhanced Expression of BvgS

**FIGURE 2:**
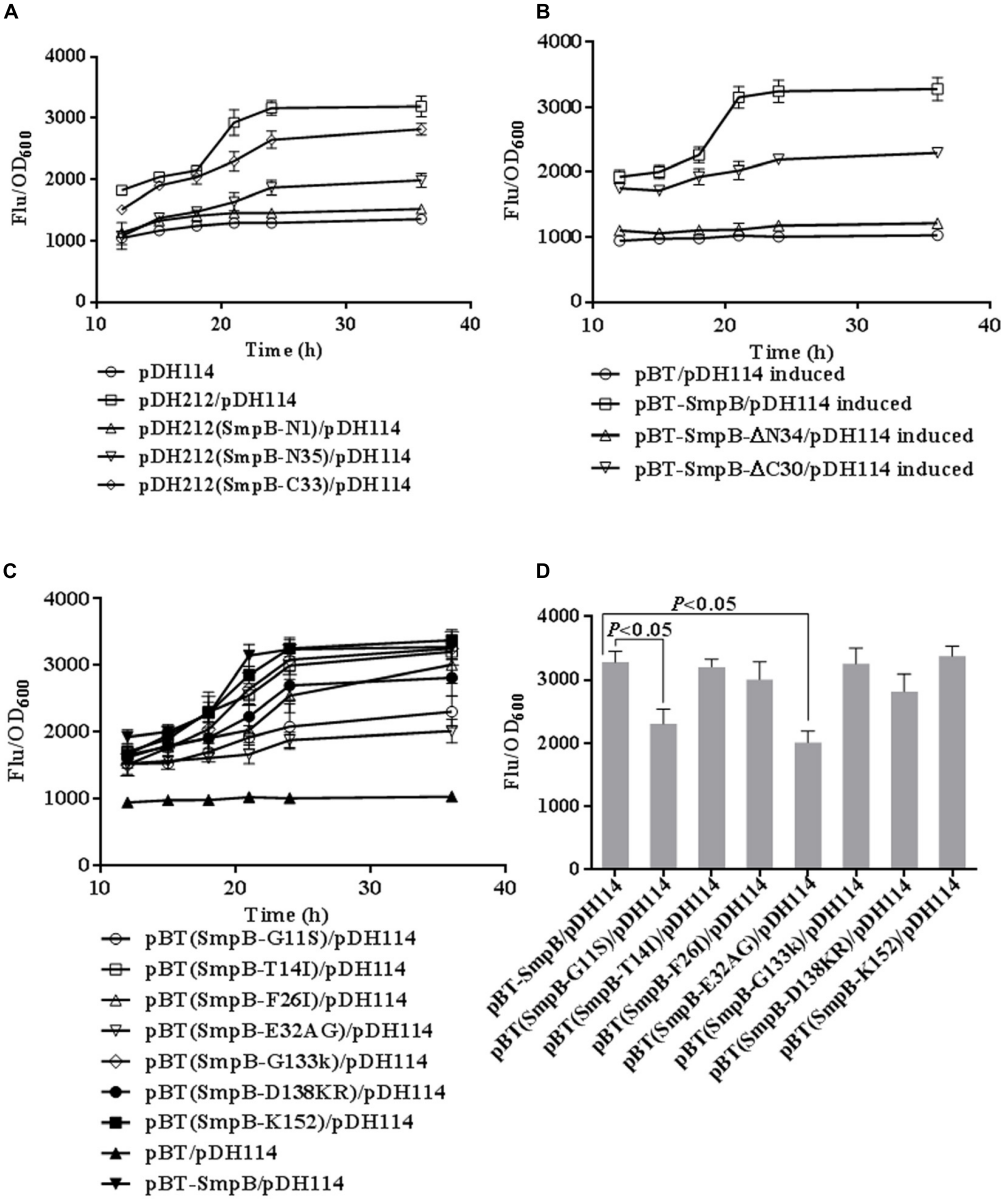
**Defining functional regions and crucial sites in SmpB protein. (A)** Time courses of relative fluorescence in *E. coli* Δ*tmRNA-smpB* co-transformed pDH114 with the mutants derived from pDH212. The fluorescence of cells was measured and normalized to corresponding OD_600_. Error bars represented SD of triplicated cultures. The mutants were introduced stop codons at 1st, 35th of N- and 33rd of C-terminal SmpB, designated as SmpB-N1, SmpB-N35, SmpB-C33, separately. **(B)** Time courses of relative fluorescence in *E. coli* two-hybrid reporter strains co-transformed pDH114 with the truncations. The mutants were truncated at 34th of N- and 30rd of C-terminal SmpB, designated as SmpB-ΔN34, SmpB-ΔC30, respectively. The cultures were induced by 1 mM IPTG at 37°C. **(C)** Time courses of relative fluorescence in *E. coli* two-hybrid reporter strains co-transformed pDH114 with the mutants. The mutants were constructed by substituting alanines for G11S, T14I, F26I, E32AG, G133K, D138KR, designated as SmpB-G11S, SmpB-T14I, SmpB-F26I, SmpB-E32AG, SmpB-G133K, and SmpB-D138KR, respectively. SmpB-K152 was mutated by substituting proline for lysine. **(D)** Column graph comparing the relative fluorescence in mutants at 24 h IPTG induction.

To investigate the effect of the N- and C- terminus regions of SmpB on the expression of *bvgS*, stop condon TAA was introduced to the 1st, or 35th amino acid of N- or the 33rd amino acid of C- terminus of SmpB in pDH212. Each resulting mutant SmpB-N1, SmpB-N35, or SmpB-C33 was co-transformed with pDH114 into *E. coli* Δ*tmRNA-smpB*. SmpB-N1 or SmpB-N35 co-transformant increased fluorescence intensity slightly compared with the negative control which only endowed with pDH114, while SmpB-C33 co-transformant improved eGFP expression but not as efficient as the positive control which endowed with pDH114 and pDH212 (**Figure 2A**). Next the truncated mutant, pBT-SmpBΔN34 or pBT-SmpBΔC30 was co-transformed with pDH114 into *E. coli* two-hybrid reporter strain. Consistently, pBT-SmpBΔN34 co-transformant increased fluorescence intensities slightly compared with the negative control which endowed with pDH114 and pBT, while pBT-SmpBΔC30 co-transformant improved eGFP expression but not as efficient as the positive control which endowed with pDH114 and pBT-SmpB (**Figure 2B**).

To investigate which amino acid at N- and C- terminus of SmpB was more important, a series of point-mutations were constructed (**Table 1**) and co-transformed with pDH114. After the cells were induced with 20 mM IPTG, the relative fluorescence intensities were analyzed at different time points (**Figure 2C**). The results showed that mutation at G11S or E32AG diminished the fluorescence intensities significantly (*p* < 0.05) at 24 h, indicating that these two amino acid sites were essential for SmpB activity.

### SmpB could Bind to the Promoter of *bvgS*

**FIGURE 3:**
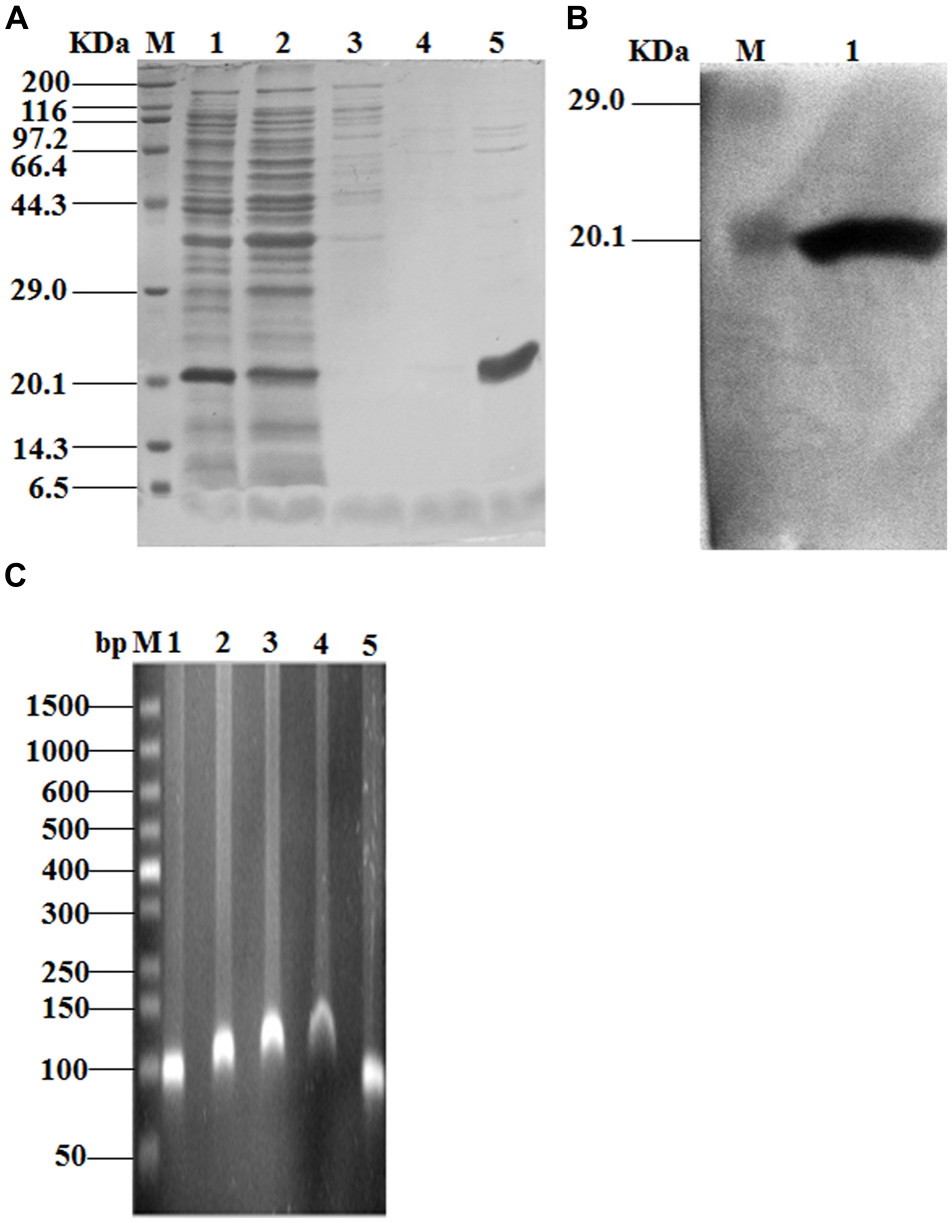
**DNA binding studies of SmpB and the promoter region of *bvgS*. (A)** Representative SDS-PAGE of elution of His_6_ tagged SmpB protein under elevated imidazole concentrations. Cell lysate (lane 1), flow through (lane 2), 50, 100, 250 mM imidazole (lane 3–5), respectively. **(B)** Western blot analysis of purified SmpB using a-His antibodies as probes. Purified SmpB (lane 1). **(C)** Agarose gel retardation assay of SmpB and the promoter region of *bvgS*. Promoter only (lane 1), the molar ratios of promoter to SmpB were 1:2, 1:4, 1:6 (lane 2–4), respectively. The molar ratio of promoter to BSA (1:6) was chosen as the negative control (lane 5).

In order to determine whether SmpB had DNA binding property, SmpB protein was purified to 95% homogeneity (**Figure 3A**) and verified by Western blot (**Figure 3B**) using His-tag antibodies. AGRA was performed for the analysis of SmpB binding to the *bvgS* promoter region. When the protein amount was increased, more of the ∼90 nt DNA fragment was bound to SmpB (**Figure 3C**), resulting in slower migration of DNA. By contrast, *yafJ* promoter region which was neighbor to *smpB* did not show the lower migration when elevating SmpB concentrations (Supplementary Figure S1).

### SmpB Positively Regulated *bvgS* Expression at the Transcriptional Level

**FIGURE 4:**
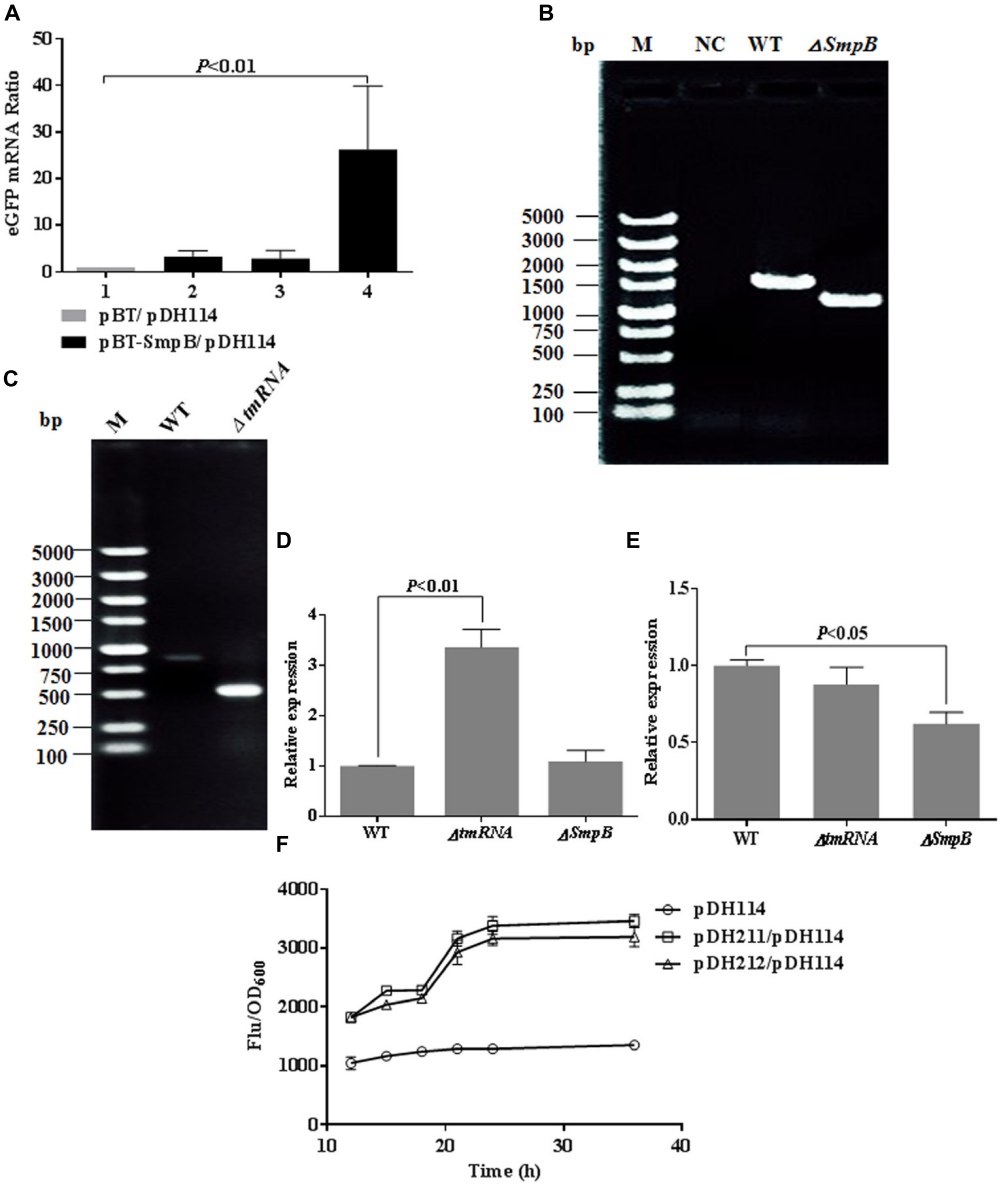
**Small protein B positively regulated BvgS expression at the transcriptional level. (A)** Comparative qRT-PCR analyses of eGFP mRNA. Relative expression was normalized to 16s RNA. The cells transformed with pBT were used as negative control (lane 1), and the cells transformed with pBT-SmpB were incubated under 1 mM IPTG induction at 37°C and collected at 12, 20, 32 h for transcriptional quantification (lane 2–4). **(B)** PCR-based verification of *smpB* knockout in *Aeromonas veronii*. **(C)** PCR-based verification of *tmRNA* knockout in *A. veronii*. **(D)** Comparative qRT-PCR analyses of *bvgS* mRNA. Relative expression was normalized to 16S RNA. The wild type and knockout strains of *A. veronii* were cultured at 37°C for 10 h and harvested for transcriptional quantification. **(E)** Comparative qRT-PCR analyses of *bvgS* mRNA. Relative expression was normalized to 16s RNA. The wild type and knockout strains of *A. veronii* were cultured at 37°C for 24 h and harvested for transcriptional quantification. **(F)** Time courses of relative fluorescence in *E. coli* Δ*tmRNA-smpB* co-transformed pDH114 with pDH211 or pDH212. The construct pDH211 possessed tmRNA and SmpB expression, and pDH212 held SmpB only.

Two methods were used to verify that SmpB upregulated *bvgS* expression at transcriptional level. First, *E. coli* Δ*tmRNA-smpB* were co-transformed with pDH114 and pBT-SmpB. Cells were harvested at 12, 20, and 32 h, and qRT-PCR was employed to detect eGFP mRNA alterations. The eGFP mRNA was elevated about 25-fold in comparison with that of control at 32 h (**Figure 4A**).

Next, *A. veronii* Δ*smpB* and Δ*tmRNA* mutants were selected and confirmed by PCR (**Figures 4B,C**). The expected 1.2 kb band was amplified from *smpB* knockout mutant compared with 1.5 kb band from wild type using primers F13/R13 (**Figure 4B**). Likewise, the expected 0.5 kb band was amplified from *tmRNA* knockout mutant compared with 0.9 kb band from wild type using primers F16/R16 (**Figure 4C**). The *bvgS* mRNA was analyzed after 10 and 24 h incubation, representing exponential and stationary phases, respectively. At 10 h cultivation, the ratio of *bvgS* mRNA in Δ*tmRNA* mutant increased about threefold compared with that in wild type and Δ*smpB* mutant, wherein the latter two remained the same (**Figure 4D**). In contrast, at 24 h cultivation, the ratio of *bvgS* mRNA in Δ*smpB* mutant reduced by about 50% compared with that of wild type and Δ*tmRNA* mutant, wherein the latter two remained the same proportionately (**Figure 4E**), suggesting that SmpB regulated *bvgS* expression only in stationary phase. Besides that, the eGFP fluorescence intensity of pDH211 carrying tmRNA and SmpB was equal to that of pDH212 carrying SmpB (**Figure 4F**), demonstrating only SmpB could upregulate eGFP production under the control of *bvgS* promoter, not tmRNA.

### SmpB Knockout Affected the Salt Tolerances in *A. veronii* C4

**FIGURE 5:**
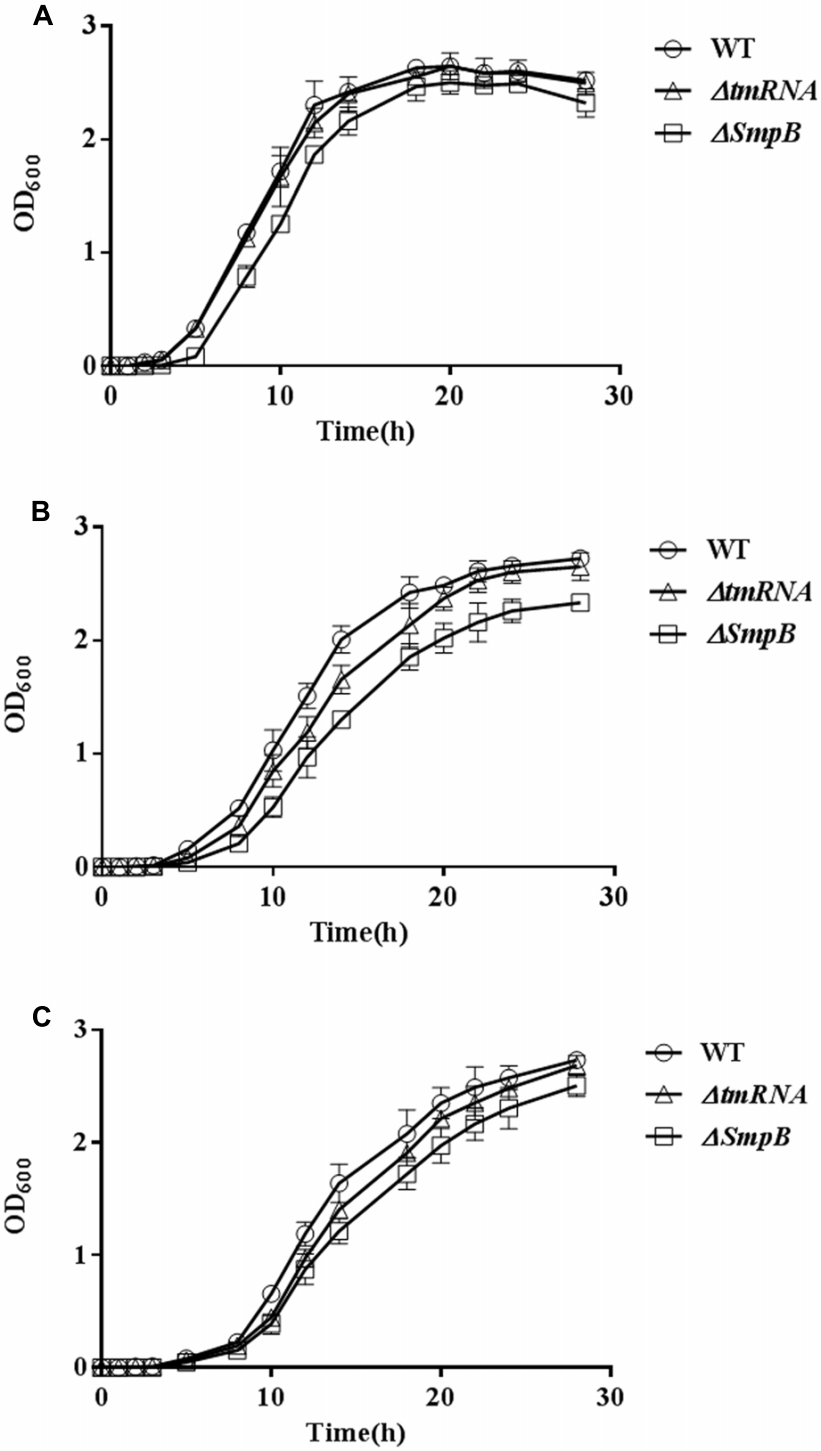
**Small protein B knockout affected the salt tolerances. (A)** Comparison of bacterial growth in M9 medium. Cells were grown at 37°C in M9 medium, and the growth rate of each strain was monitored by measuring OD_600_. Error bars represented SD of three independent cultures. **(B)** Comparison of bacterial growth in M9 medium supplemented with 0.3 M KCl. **(C)** Comparison of bacterial growth in M9 medium supplemented with 0.3 M KCl and 50 mM MgSO_4_.

To evaluate the effect of *smpB* knock-out on biological processes in *A. veronii*, the growth was measured in M9 medium or M9 supplemented with 0.3 M KCl for 24 h (Hiroaki et al., 2011). Only slight differences of growth were observed in normal M9 medium (**Figure 5A**). However, *A. veronii* Δ*smpB* were more susceptible to salt stress (0.3 M KCl) as evidenced by significantly slower growth in comparison with wild type *A. veronii*, while *A. veronii* Δ*tmRNA* fell in between them (**Figure 5B**). Consistently, when 50 mM MgSO4 was added into the culture, the same tendencies were observed (**Figure 5C**).

## Discussion

Great advances have been made in understanding the mechanism of *trans*-translation, which is mediated by tmRNA and SmpB for translational surveillance and ribosome rescue. In brief, the ribosomes do not read through the 3^′^ end of a damaged mRNA when transcription error and translational frameshifting are occurred, follows by the quaternary complex composed of ananyl-tmRNA, SmpB protein, EF-Tu, and GTP entering the stalled ribosomes to release the subunits, and ultimately the proteases and ribonuclease target the nascent polypeptides and mRNAs for degradation, independently (Keiler, 2008). The C-terminus of SmpB protein is crucial to the *trans*-translation process, which functions as a helix on the ribosome to promote tmRNA accommodation (Miller et al., 2011). Specifically, resides D(138)KR and K151 in the C-terminus have been identified essential for this step in *E. coli* (Miller et al., 2011).

In this study, our results showed that the initial 34 residues of N-terminal SmpB played essential roles, and the last 30 residues of C-terminus were also affected on the increase of fluorescence (**Figure 2B**). Surprisingly, conserved residues D(138)KR and K151 in C-terminus of SmpB played little effect on the upregulation of BvgS, compared with residues G11S and E32AG in the N-terminus of SmpB (**Figures 2C,D**). These studies indicated that SmpB functioned in a different way between *trans*-translation system and BvgS regulation in bacteria. SmpB could bind to the promoter of *bvgS* gene (**Figure 3C**) and upregulated the expression at transcriptional level (**Figure 4A**), presenting that SmpB played not only as a tmRNA binding protein but also a transcription activator.

Our work also indicated that tmRNA and SmpB might play distinct regulation modes for *bvgS* expression in exponential and stationary phases (**Figures 4D,E**). To confirm the hypothesis, the relative fluorescence of the cells conferring both SmpB and tmRNA expression was compared with that of the cells conferring only SmpB expression (**Figure 4F**), indicating that tmRNA expression could not promote BvgS expression at transcriptional level in stationary phase.

The *smpB* knockout possessed the lowest growth rate under salt tolerance in *A. veronii*, while tmRNA knockout suffered the moderate effects (**Figure 5B**), also manifesting that tmRNA and SmpB performed different functions beyond *trans*-translation in *A. veronii*. Next we wondered whether BvgS inhibition could adjust to obtain the same growth rate in wild type, Δ*smpB*, and Δ*tmRNA* because BvgS was downstream of SmpB in the regulatory pathway. Since few studies were performed on BvgS/RR TCS in *A. veronii*, while a lot of researches were finished up in *B. pertussis*, the *bvgS* amino acids identities were at 40% between *A. veronii* and *B. pertussis*, resulting us to assume that BvgS inhibition of *B. pertussis* was adopted for references in *A. veronii*. Previous studies revealed that BvgS functions were inhibited in the presence of sulfate anion or nicotinic acid in *B. pertussis* (Miller et al., 1992; Veal-Carr and Stibitz, 2005), so inferred BvgS inhibitor (50 mM MgSO_4_) was supplemented for the observation of bacterial growth under salt tolerance (0.3 M KCl) in *A. veronii*. However, the growth tendency remained similar before and after adding MgSO_4_ among three strains (**Figure 5C**), suggesting that BvgS function might not be inhibited using 50 mM MgSO_4_ in *A. veronii*.

In summary, SmpB protein, which considered as the major component in *trans*-translation system, was identified to upregulate the expression of *bvgS*, which was a sensor protein involved in signal transduction in *A. veronii*. SmpB could bind to the promoter region of *bvgS* and activate the transcription process. We believe this is the first report on SmpB functions besides *trans*-translation in *A. veronii*.

## Conflict of Interest Statement

The authors declare that the research was conducted in the absence of any commercial or financial relationships that could be construed as a potential conflict of interest.
