# Supplementary material for: Small protein B upregulates sensor kinase bvgS expression in Aeromonas veronii
Source: Front Microbiol. 2015 Jun 16;6:579. doi: 10.3389/fmicb.2015.00579 (PMC4468919; doi:10.3389/fmicb.2015.00579)
Supplement: Supplementary file 1 [file Table_1.DOC]

| **Supplementary Table 1. Primers used in this study.** | | |
| --- | --- | --- |
| **Plasmid** | **Sequence (5´-3´)** | **Enzyme** |
| pDH114 | F1:5´-GA*AGATCT*AACGTTTGGAAAGGGCCACC-3´  R1:5´-CCTCGCCCTTGCTCACCATGAAGAGACGATTACATTGACTGGGCAT-3´  F2:5´-ATGCCCAGTCAATGTAATCGTCTCTTCATGGTGAGCAAGGGCGAGG-3´  R2:5´-GCCG*CGGCCG*TTACTTGTACAGCTCGTCCAT-3´ | *Bgl* II  *Eag* I |
| pDH211  pDH212  pDH213 | F3:5´-CATG*CCATGG*agctcaccagatcgcggaat-3´  R3:5´-CCG*CTCGAG*TGGTGGAGCTGGGGGGATT-3´  F4:5´-CATG*CCATGG*agctcaccagatcgcggaat-3´  R4:5´-CCG*CTCGAG*TTAGCCGCGATGCTTGTTCT-3´  F5:5´-CATG*CCATGG*agctcaccagatcgcggaat-3´  R5:5´-CCG*CTCGAG*GACGGGCGATTTCCGGCAA-3´ | *Nco* I  *Xho* I  *Nco* I  *Xho* I  *Nco* I  *Xho* I |
| pDH214 | F6:5´-CATG*CCATGG*TGGCTGTGCAGGTCGTAAAT-3´  R6:5´-CCG*CTCGAG*TCACGATGCGGCCGCTCG-3´ | *Nco* I  *Xho* I |
| pET-SmpB | F7:5´-CATGCCATGGGCAGCAAAAAAAACAGTAAA  AACAAAGC -3´  R7:5´-CCGCTCGAGGCCGCGATGCTTGTTCTTCAT-3´ | *Nco* I  *Xho* I |
| pBT-SmpB  pBT-SmpB  △N34  pBT-SmpB  △C30 | F8:5´-CG*GAATTC*CATGAGCAAAAAAAACAGTAAAAAC-3´  R8:5´-GA*AGATCT*TTAGCCGCGATGCTTGTTC-3´  F9:5´-CG*GAATTC*CCTGTCCCTGCAAGGGTGG-3´  R9:5´-GA*AGATCT*TTAGCCGCGATGCTTGTTC-3´  F10:5´-CG*GAATTC*CATGAGCAAAAAAAACAGTAAAAAC-3´  R10:5´-GA*AGATCT*TTACTTCACCAGGCCGATCTCG-3´ | *Eco*R I  *Bgl* II  *Eco*R I  *Bgl* II  *Eco*R I  *Bgl* II |
| pRE-△SmpB | F11:5´- CGG*GGTACC*CATGTAGTCATCGCCAGCCT -3´  R11:5´-GATGCTTGTTCTTCATGATGCGCTTCTCTTCGATGAAGTATTCG -3´  F12:5´-CGAATACTTCATCGAAGAGAAGCGCATCATGAAGAACAAGCATC -3´  R12:5´- AA*GAGCTC*TCCACACGATCCTGTGAAGG-3´  F13:5´- AGCTCACCAGATCGCGGAATAC -3´  R13:5´- TGGTGGAGCTGGGGGGATTT-3´ | *Kpn* I  *Sac* I |
| pRE-△tmRNA | F14：ATAGAT*AAGCTT*acccgcacccgcaaactg  R14：GC*TCTAGA*AAGTTGTTAATTGCGATTGTACG  F15：GC*TCTAGA*aacgtttggaaagggccacC  R15：AT*CCCGGG*CAGACAGATCAAACTCGTCTTC  F16：GCGCAGCTGGCTCTTCAGG  R16：TCCAACCAGTTTGATATCGG | *Hin*d III  *Xba* I  *Xba* I  *Xma* I |
| **Mutant** | **Sequence (5´-3´)** | |
| pDH212  (SmpB-N1) | F17:5´-TTGCCGGAAATCGCCCGTC**TAA**AGCAAAAAAAACAGTAAAAACAAAG-3´  R17:5´-CTTTGTTTTTACTGTTTTTTTTGCT**TTA**GACGGGCGATTTCCGGCAA-3´ | |
| pDH212  (SmpB-N35) | F18:5´-AAGAGAAGATCGAAGCGGGT**TAA**TCCCTGCAAGGGTGGGAAG-3´  R18:5´-CTTCCCACCCTTGCAGGGA**TTA**ACCCGCTTCGATCTTCTCTT-3´ | |
| pDH212  (SmpB-C33) | F19:5´-CAAGGTCGAGATCGGCCTG**TAA**AAGGGCAAGAAAGAGCACGA-3´  R19:5´-TCGTGCTCTTTCTTGCCCTT**TTA**CAGGCCGATCTCGACCTTG-3´ | |
| pBT  (SmpB-G11S) | F20:5´-CAAAAAAAACAGTAAAAACAAAGCC**GCAGCA**AGCACCATTGCACTCAACAGA-3´  R20:5´-TCTGTTGAGTGCAATGGTGCT**TGCTGC**GGCTTTGTTTTTACTGTTTTTTTTG-3´ | |
| pBT  (SmpB-T14I) | F21:5´-AACAAAGCCGGGTCCAGC**GCAGCA**GCACTCAACAGAACCGCG-3´  R21:5´-CGCGGTTCTGTTGAGTGC**TGCTGC**GCTGGACCCGGCTTTGTT-3´ | |
| pBT  (SmpB-F26I) | F22:5´-ACCGCGCGCCACGAATAC**GCAGCA**GAAGAGAAGATCGAAGCGG-3´  R22:5´-CCGCTTCGATCTTCTCTTC**TGCTGC**GTATTCGTGGCGCGCGGT-3´ | |
| pBT  (SmpB-E32AG) | F23:5´-ATACTTCATCGAAGAGAAGATC**GCAGCGGCA**CTGTCCCTGCAAGGGTGG-3´  R23:5´-CCACCCTTGCAGGGACAG**TGCCGCTGC**GATCTTCTCTTCGATGAAGTAT-3´ | |
| pBT  (SmpB-G133K) | F24:5´-CGAGATCGGCCTGGTGAAG**GCAGCA**AAAGAGCACGACAAGCGCGA-3´  R24:5´-TCGCGCTTGTCGTGCTCTTT**TGCTGC**CTTCACCAGGCCGATCTCG-3´ | |
| pBT  (SmpB-D138KR) | F25:5´-TGAAGGGCAAGAAAGAGCAC**GCAGCAGCA**GAAGATACCAAGGCCCGCG-3´  R25:5´-CGCGGGCCTTGGTATCTTC**TGCTGCTGC**GTGCTCTTTCTTGCCCTTCA-3´ | |
| pBT  (SmpB-K152) | F26:5´-CGCGAGTGGGATCGGGAG**CCT**GCCCGCATCATGAAGAACAA-3´  R26:5´-TTGTTCTTCATGATGCGGGC**AGG**CTCCCGATCCCACTCGCG-3´ | |
| **Gene** | **Primers for AGRA** | |
| *bvgS* | F27:5´-CATGTCGCGGCCCAATATTG-3´  R27:5´-CAGAAGGAATCCAACAGAATTG-3´ | |
| ***yafJ*** | F28:5´- ATGAAAGTGATCGCCCTGAC -3´  R28:5´- ATCCGGCAGACTCCTTTCATG -3´ | |
| **Gene** | **Primers for Real-time PCR** | |
| *eGFP* | F29:5´-ATGGTGAGCAAGGGCGAG-3´  R29:5´-CTTGCCGTAGGTGGCATC-3´ | |
| *bvgS* | F30: 5´-GCACGTCTCTCTCCATCAAT-3´  R30: 5´-CATGTTGGCTAGTCGTAGTG-3´ | |
