# Supplementary material for: Small protein B upregulates sensor kinase bvgS expression in Aeromonas veronii
Source: Front Microbiol. 2015 Jun 16;6:579. doi: 10.3389/fmicb.2015.00579 (PMC4468919; doi:10.3389/fmicb.2015.00579)
Supplement: Supplementary file 2 [file Image_1.PDF]

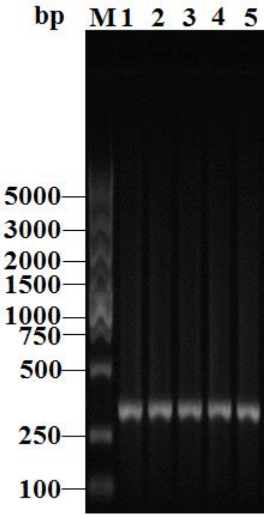

Supplementary FIGURE 1 | Agarose gel retardation assay of SmpB and the promoter region of *yafJ*. Promoter only (lane 1), the molar ratios of promoter to SmpB were 1:2, 1:4, 1:6 (lane 2-4), respectively. The molar ratio of promoter to BSA (1:6) was chosen as the negative control (lane 5).
